# Methods for comprehensive chromosome screening of oocytes and embryos: capabilities, limitations, and evidence of validity

**DOI:** 10.1007/s10815-012-9727-9

**Published:** 2012-03-14

**Authors:** Nathan R. Treff, Richard T. Scott

**Affiliations:** 1 Reproductive Medicine Associates of New Jersey, Morristown, NJ 07960 USA; 2 Department of Obstetrics Gynecology and Reproductive Science, University of Medicine and Dentistry of New Jersey, Robert Wood Johnson Medical School, New Brunswick, NJ 08901 USA; 3 Department of Genetics, Rutgers-The State University of New Jersey, Piscataway, NJ 08854 USA

**Keywords:** Comprehensive chromosome screening, Preimplantation genetic screening, Microarray, Comparative genomic hybridization, Randomized controlled trial

## Abstract

Preimplantation aneuploidy screening of cleavage stage embryos using fluorescence in situ hybridization (FISH) may no longer be considered the standard of care in reproductive medicine. Over the last few years, there has been considerable development of novel technologies for comprehensive chromosome screening (CCS) of the human genome. Among the notable methodologies that have been incorporated are whole genome amplification, metaphase and array based comparative genomic hybridization, single nucleotide polymorphism microarrays, and quantitative real-time PCR. As these methods become more integral to treating patients with infertility, it is critical that clinicians and scientists obtain a better understanding of their capabilities and limitations. This article will focus on reviewing these technologies and the evidence of their validity.

## Comprehensive Chromosome Screening (CCS) methodologies

The concept of preimplantation genetic screening (PGS) for chromosomal abnormalities to improve clinical outcomes in patients undergoing in vitro fertilization (IVF) is based on the observation that aneuploidy represents the leading genetic cause of miscarriage [1] and is likely the most prevalent genetic abnormality in human embryos [2]. In addition, conventional embryo selection methodologies, namely using morphological and developmental criteria, are not sufficient to identify which embryos are chromosomally normal [3]. Despite sound principles for the use of PGS, clinical outcomes from FISH based PGS, the most commonly used technology, have been disappointing [4].

One of the key limitations of FISH based preimplantation aneuploidy screening is the inability to simultaneously evaluate all 24 chromosomes found in human cells (chromosome 1–22, X and Y). The development of technologies for single cell whole genome amplification [5–9] has now led to a number of methodologies for comprehensive screening of all 24 chromosomes in preimplantation embryos and polar bodies. These CCS technologies include metaphase comparative genomic hybridization (mCGH) [10–12], array (a)CGH [5, 6, 13, 14], and single nucleotide polymorphism (SNP) arrays [15–19]. More recently, a quantitative real time (q)PCR approach was developed that does not require whole genome amplification [20]. Despite similar applications (i.e. 24 chromosome aneuploidy screening) these various methods of CCS possess unique capabilities and limitations (Table 1).

**Table 1:** Comparison of methods for preimplantation CCS

Reported characteristic	CCS method				
	mCGH	short mCGH	aCGH	SNP array	qPCR
Accuracy^a^	NR	100% [22]	NR	94 [16] -99% [15]	97% [20]
Consistency between PB and oocyte	76 [81]-88% [82, 83]	NR	94% [60]	NR	NR
Minimum turn-around time	72 h	24 h	12 h [60]	24 h [16]	4 h [20]
Stages of biopsy eligible for fresh ET	oocyte	oocyte, cleavage	oocyte, cleavage	oocyte, cleavage	oocyte, cleavage, blastocyst
Number of probes	NA	NA	2–32 K	262–370 K	NR
Reported minimum detectable imbalance	10 Mb [29]	100 Mb [30]	2.5 Mb [32]	1.7 Mb [28]	NR
Direct monogenic disease screening^b^	-	-	-	+	NR
Uniparental disomy screening	-	-	-	+	NR
Contamination screening	-	-	-	+	NR
Origin of aneuploidy screening	-	-	-	+^c^	NR

^a^Accuracy predictions based on reported analysis of cell lines

^b^mCGH, short mCGH, and aCGH have been reported with parallel but indirect monogeneic disease screening

^c^Not all SNP array methods have validated origin of aneuploidy predictions

*NR* Not reported, *NA* Not applicable

One important consideration is the amount of time required to complete the analysis. While rapid conventional mCGH methods have been developed [21–23], the most widely used methods aren’t suitable for cleavage stage biopsy and fresh embryo transfer. However, mCGH results are typically available in time for a fresh embryo transfer when applied to polar body biopsy [12, 24]. Currently, most groups utilize aCGH instead of mCGH in clinical trials and commercial activities as aCGH allows for greater throughput, higher resolution and more rapid analyses. SNP arrays can also be used on blastomeres in time for fresh embryo transfer [16, 25] and additional time savings are possible through the use of qPCR methods from which results are available within 4 h of the biopsy allowing fresh blastocyst transfer after trophectoderm biopsy [20, 26].

Although aCGH and SNP array technology both involve an array of DNA based probes, these methods are not equivalent. For example, among the commonly used arrays for PGS, the BlueGnome (Cambridge, UK) bacterial artificial chromosome (BAC) arrays possess approximately 2,000 to 5,000 DNA probes across 24 chromosomes [27], the Affymetirx (Santa Clara, CA) NspI SNP array possesses approximately 262,000 probes [15, 28], and Illumina (San Diego, CA) arrays used typically possess approximately 300,000 to 370,000 probes [16–18]. Although the number of probes included on each of these arrays does not necessarily correlate with the level of accuracy, it does influence the level of genomic resolution provided by each method. Furthermore, BAC array and SNP array probe numbers may not be directly compared since relative performance of probes on each array type can impact resolution. While it is clear that whole chromosome aneuploidy represents the most common and clinically relevant genetic abnormality in human embryos, many groups have developed CCS technologies capable of identifying smaller deletions and duplications. This capability is particularly important for patients carrying a balanced translocation since clinically significant imbalances of smaller segments of the chromosomes (segmental aneusomy) involved are often produced during meiosis. A number of CCS methods have been applied to evaluating embryos from translocation carrier patients including mCGH with a resolution of 10–20 Mb [29] and 25–100 Mb [30], aCGH with a resolution of 2.8 Mb [31] and 2.5 Mb [32], and SNP arrays with a resolution of 2.4 Mb [33] and 5 Mb [34]. Despite these reported abilities to detect small imbalances, the ability to predict de novo deletions and duplications in embryos from patients without a known translocation has typically not been claimed. However, a method combining aCGH and SNP array technologies suggested the ability to detect a 1.7 Mb deletion in single cells [28] and to predict de novo deletions and amplifications in human embryos [35]. Estimates of the prevalence of such imbalances in human embryos by this methodology are high given their rarity in clinically recognized pregnancies [36, 37] and may need to be confirmed by alternative methodologies.

Another key difference between array based platforms is the way in which copy number is assigned for each chromosome. For example, aCGH involves differential labeling and mixing of biopsy DNA with control DNA prior to hybridization and interpretation of ratios of red and green (two-color) fluorescence upon completion [38]. In contrast, Affymetrix SNP arrays involve hybridization of only biopsy DNA (single color) followed by computational comparison of signal intensities to those obtained on separate control DNA hybridized arrays (*in silico* controls) [39]. The SNP single color array approach has the distinct advantage of evaluating the test sample against a large number of control samples (not just 1). This could help avoid inconsistencies from control sample specific natural variations in the human genome. However, aCGH platforms are typically designed with probes that avoid regions of the genome with polymorphic copy number variations. The aCGH two-color approach has the advantage of paired comparison to a control sample produced during the same timeframe and with the same lot of reagents used for the test sample. This could help control for fluctuations in laboratory components used in the process over time.

While CGH methodologies provide an assessment of chromosomal copy number, SNP arrays and qPCR can also provide genotypic information which can be used for the assessment/diagnosis of multiple other clinical factors. These include single gene disorders, uniparental disomy (UPD), loss of heterozygosity (LOH), DNA fingerprinting and determination of the parental and cell division origins of aneuploidy. Although simultaneous aneuploidy (using mCGH [24, 40, 41] and SNP arrays [18, 42]) and single gene (using conventional PCR) analysis has been reported, SNP array based haplotype methodologies provide the ability to avoid the time and expense of preparing PCR based family specific informative markers of the mutation but may be limited when additional family members are not available to define haplotype phases. A recent study illustrated the use of SNP arrays to predict inheritance of monogenic disease through haplotype based analysis [17] and has applied the methodology clinically [43]. Another group presented a similar application of SNP arrays with results confirmed from antenatal analysis of 3 pregnancies [44].

A number of groups using SNP arrays have used the technology to predict the parental and cell division origins of embryonic aneuploidy. One recent publication indicated an accuracy of 100% for predicting the parental origin of monosomy (26/26 male embryo X chromosomes identified as maternal), and 50% for trisomy [35]. Treff et al. (2008) [45] not only modeled monosomy by evaluating the X chromosome from male embryos, but also used embryos with trisomies known to originate from maternal meiosis (as a result of having evaluated the polar bodies from the same oocyte that the embryo was derived from) and demonstrated 100% accuracy for identifying both monosomy and trisomy parental origin of aneuploidy from SNP array data alone [45]. Other groups have described results of predicting the parental origin of aneuploidy but have yet to report levels of accuracy from controls [16, 46]. Furthermore, some methods have been applied to distinguish meiotic from mitotic aneuploidy by investigating the patterns of crossover and haplotype inheritance [16, 47]. Validation of these methods in single cells also remains to be presented but could represent an important advancement towards improving our understanding of the origin and etiology of preimplantation aneuploidy. Further research into the accuracy of predicting the cell division origins of aneuploidy in human embryos by SNP array technology are of particular clinical importance since they have been proposed to serve as a means to justify the transfer of embryos with aneuploid results [48].

Although uniparental isodisomy may represent an extremely rare event, the possibility of detection has been validated using SNP array technology through loss of heterozygosity (LOH) analysis [49]. LOH analysis can also be useful in confirming the presence of a monosomy identified through copy number based analyses [15]. In contrast, the ability to identify uniparental heterodisomy has yet to be validated but is theoretically possible given the ability to predict the parental origin of aneuploidy. Finally, DNA fingerprinting from SNP array data provides an opportunity to prevent misdiagnosis from contamination and also to identify and track which embryo implanted after multiple embryo transfer [50]. The later of these fingerprinting applications provides a unique opportunity to perform very well controlled paired analyses of putative markers of, or the impact of interventions on the reproductive potential of sibling embryos [51].

Despite the numerous differences between platforms of CCS, very little is known about the comparative performance of each laboratory specific methodology. Still, comparative data may be critical to a better understanding of the limitations and capabilities of various CCS methodologies and to providing patients with the best possible care. Some comparative studies of methodologies within groups have been performed. For example, mCGH and SNP arrays [52], and qPCR and SNP arrays [26] have indicated similar levels of performance. In contrast, a comparative study between aCGH and SNP arrays indicated aCGH methodology to have a significant decrease in diagnostic accuracy compared to SNP arrays [53]. However, a recent study found that other platforms for aCGH (BlueGnome) and SNP arrays (Illumina) were highly concordant [54]. Additional comparative studies, not just across platforms but also across laboratories, remain critical to identifying the most accurate methodologies of CCS.

## Comparison of CCS and FISH

**Fig. 1:**
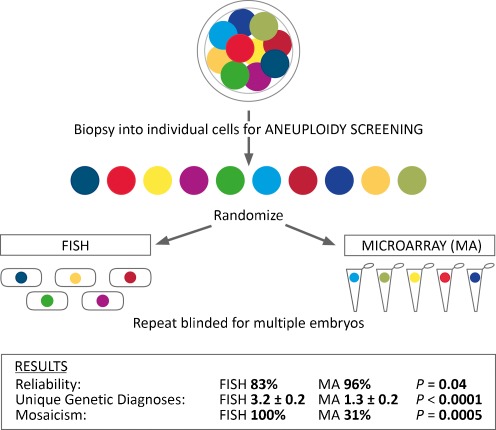
A prospective randomized blinded study designed to compare the level of reliability and consistency of 2 methods of aneuploidy screening. Arrested cleavage stage embryos can be dispersed into individual blastomeres and then randomly assigned to analysis by either of 2 methods of analysis (i.e. FISH and SNP microarray). By including more than one embryo in the randomization, the embryo of origin of each blastomere can also remain blinded, thereby avoiding the potential bias from knowing that 2 blastomeres originated from the same embryo. Results can demonstrate which method provided the most reliable and consistent diagnosis. Adapted from Treff et al. [55], and used with permission from Oxford University Press

With the development of new methods for aneuploidy screening such as CCS, studies of the putative limitations of FISH based methods have begun to emerge. One of these studies involved a novel prospective randomized blinded analysis of multiple single blastomeres from the same cleavage stage embryos using either FISH or SNP array based CCS (Fig. 1) [55]. Randomization of blastomeres from the same embryo to analysis by either of the two methods provided a unique opportunity to distinguish between the contribution of mosaicism (biological error) or technical error by assessing the rate of discordance observed by each method. That is, the rate of discordance within each embryo should be similar for each method used since mosaicism should be equally distributed (randomly) to the two methods. However, FISH predicted 100% mosaicism while SNP arrays found only 31% mosaicism (Fig. 1) indicating that FISH is an inconsistent technology. This is particularly significant since the SNP array data included more chromosomes per cell, and more cells per embryo (as a result of significantly higher reliability of diagnosis), both of which would increase the chances of finding mosaicism in the SNP array group.

A second study comparing FISH and CCS analysis of the same embryos involved careful evaluation of the putative mechanisms of “self correction,” [49] which represents the primary explanation for discordance between cleavage stage FISH and reanalysis of the resulting blastocysts. For example, one proposed mechanism of correction is trisomy/monosomy rescue through extrusion or duplication of a trisomic or monosomic chromosome, respectively. This phenomenon can be expected to lead to uniparental disomy (UPD) in a certain percentage of blastocysts. Unlike previous studies that have used FISH to reanalyze blastocysts, originally diagnosed as abnormal on day 3, SNP array based analysis provides an opportunity to investigate the possibility of self correction resulting in UPD. The study by Northrop et al. (2010) [49] indicated that trisomy/monosomy rescue is not the major mechanism for “self correction” as 0% of the “corrected” chromosomes (*n* = 204) displayed UPD after evaluation with SNP arrays [49]. Another possible mechanism of correction is preferential segregation of abnormalities to the trophectoderm. Multiple CCS studies have now demonstrated that preferential segregation does not occur [49, 56, 57]. However, a potential limitation of interpretation from blastocyst reanalysis by CCS methodologies is the inability to detect the presence of mosaicism within a multi-cell biopsy. Northrop et al. (2010) [49] specifically validated the ability to detect a level of mosaicism of 40% within a 5-cell sample and therefore provided more weight to evidence for a lack of preferential segregation [49]. While it is still possible that complete extrusion or apoptosis of aneuploid cells may result in correction of abnormalities in the embryo upon blastulation, no evidence currently exists to support such a phenomenon.

One of the major implications of these comparative studies is that the mitotic origin of embryonic aneuploidy may have been overestimated. Indeed, studies of products of conception have found that maternal meiosis is the predominant origin of aneuploidy [58], and not mitosis as some methods of PGS have predicted. While traditional cytogenetic analysis (G-banding) may underestimate the levels of mosaicism in products of conception, recent molecular cytogenetic studies have shown that “actual” levels of mosaicism may only be higher by a marginal percentage [36, 37]. In fact, methods which predict high levels of mosaicism in embryos may need to be re-evaluated as illustrated in Fig. 1 in order to distinguish between biological phenomena and technical inconsistency. This design could also represent an important component to preclinical validation of embryonic CCS methodologies. Still, the phenomenon of mosaicism cannot be completely disregarded given observations from other studies involving CCS analysis of multiple cells from the same embryo [11, 59].

## Evidence for CCS

**Fig. 2:**
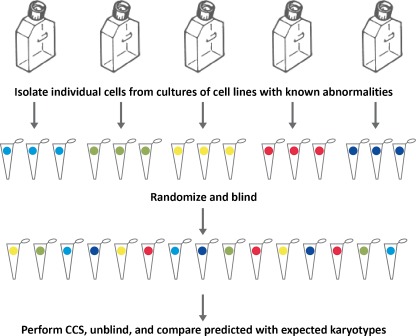
A prospective randomized blinded study designed to determine the accuracy of a single cell CCS methodology. Cell lines with previously well characterized chromosomal abnormalities can be obtained from a number of commercial suppliers such as the Corriel Cell Repository (Camden, NJ). Lines with consistent abnormalities observed in multiple evaluations by the supplier may provide the most consistent single cells in terms of possessing the expected karyotype. Single cells can be obtained, placed in PCR tubes, and randomized and blinded for analysis by CCS. Once CCS predictions are made, the origin of each cell can be unblinded to evaluate the consistency with the expected karyotype and the accuracy of the CCS methodology can be determined

Given the inconsistency and poor negative predictive value of cleavage stage FISH, new technologies such as CCS should be more carefully evaluated. Moreover, FISH based re-analysis of CCS evaluated embryos may not represent a good approach to validate new CCS methods since FISH has been shown to be an inconsistent methodology itself. Alternatively, randomized blinded analysis of single cells from cell lines with known abnormalities could be performed and used to evaluate the accuracy of aneuploidy predictions (Fig. 2). Another interesting study design for evaluating the accuracy of a CCS methodology was recently performed by the European Society of Human Reproduction and Embryology (ESHRE) Task Force on PGS [60]. In this blinded pilot study, polar body CCS results were obtained using aCGH and compared to the results of the corresponding oocytes for consistency, which was found to be 94% (130/138).

**Fig. 3:**
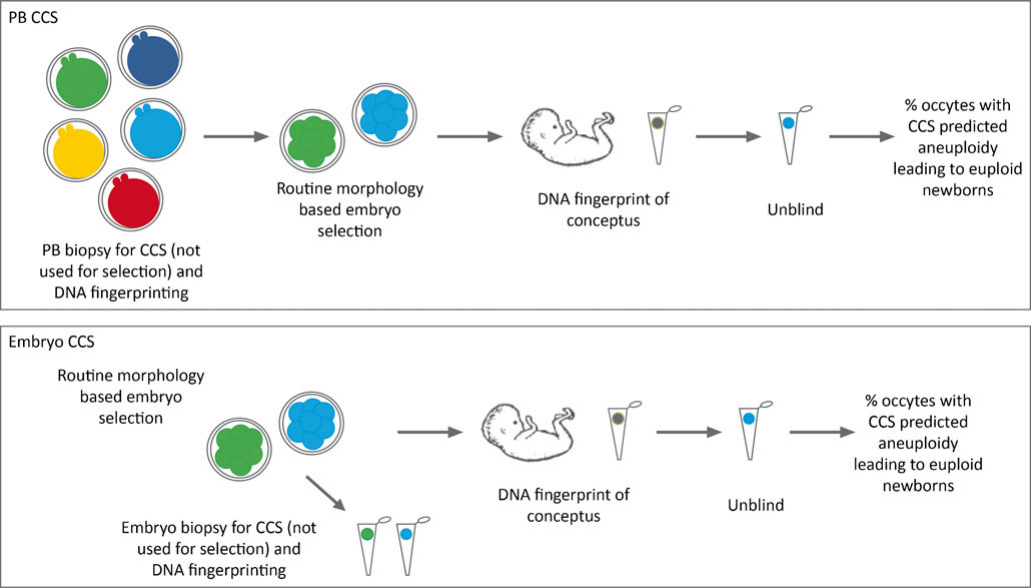
A prospective blinded non-selection study designed to determine the negative predictive value of a CCS methodology for the reproductive potential of the oocyte or embryo. Biopsies of either polar bodies from the oocyte, a blastomere from the cleavage stage embryo, or trophectoderm from the blastocyst can be performed. The best embryos can be selected for transfer based on conventional criteria and without the use of CCS results. DNA from the conceptus can be obtained and evaluated against the DNA from the original biopsies in order to determine which oocytes or embryos produced the newborns. The percentage of oocytes or embryos predicted to have possessed aneuploidy by the CCS methodology and that produced euploid newborns can be calculated. This value subtracted from 100% gives the negative predictive value and whether the CCS methodology can be used to safely discard an embryo

As previously alluded to, accumulating evidence indicates that FISH based aneuploidy screening at the cleavage stage of embryogenesis is not only inconsistent but also poorly predictive of aneuploidy in the blastocyst. Reanalysis of blastocysts indicate that nearly 60% of embryos discarded due to an abnormal cleavage stage FISH diagnosis were in fact euploid for all 24 chromosomes in all 4 evaluated sections [49]. A similar observation was recently made from reanalysis of blastocysts originally given a parental support based CCS abnormal cleavage stage diagnosis [61]. Parental support based CCS reanalysis results indicated that 43% of the blastocysts (26/61) were euploid despite an aneuploid diagnosis at the cleavage stage. It is unclear whether these discrepancies were due to biological variation or technical issues with parental support. These observations highlight the critical need for a second preclinical validation study; to determine the negative predictive value of CCS for embryonic reproductive potential [62]. Such a study could indicate whether the CCS method is capable of accurately determining the chromosomal status of embryos so that there is high confidence that embryos being discarded are truly abnormal (Fig. 3). Oocytes or embryos would be biopsied and selected for transfer using conventional morphological and developmental criteria without information from CCS. The CCS results of each biopsy would be produced only after embryo transfer had taken place. Specific outcomes of each individual oocyte or embryo would be confirmed by DNA fingerprinting [50] and the implantation and delivery from oocytes or embryos that would have been diagnosed as aneuploid by CCS could be determined. However, since some CCS methods don’t provide polymorphism information (i.e. mCGH and aCGH), additional molecular tests would be required in order to track embryo specific outcomes. This approach should be possible even for those groups using CGH methodologies given previous reports of double factor PGD [40, 41]. In the situation where polar body biopsy is performed, an alternative method of embryo tracking from polar body polymorphisms could be employed [63]. While a randomized controlled trial is necessary to evaluate the efficacy of a particular CCS method it would fail to determine the negative predictive value of the method since samples predicted to possess aneuploidy would not be transferred. This is particularly troublesome for patients when all their embryos or oocytes are predicted to be aneuploid. Without the knowledge that the negative predictive value of a method is meaningful, one will never know if implementation of the method prevented such a patient from missing out on the opportunity of having a healthy child. A prospective blinded non-selection clinical trial is therefore a critical consideration when establishing the safety of CCS methodologies.

Although a randomized controlled trial represents what most consider the pinnacle of clinical validation of efficacy, case–control studies currently represent the primary source of evidence of clinical validity for most CCS methodologies [12, 64–68]. While it is certainly encouraging to see improvements from such analyses, it should be noted that many cleavage stage FISH case–control studies illustrated significant clinical improvements [69–73] but ultimately failed in all randomized controlled trials performed to date [4]. As a result of the higher standard of evidence that randomization provides, many CCS methodologies have now initiated the process of conducting randomized controlled trials (Table 2). The results presented for one these CCS methods has indicated a significant increase in clinical pregnancy and embryo implantation rates [26].

**Table 2:** Randomized Controlled Trials of CCS

	Colorado Center for Reproductive Medicine	ESHRE Task Force on PGS	GENERA Center for Reproductive Medicine	Gene Security Network	Reproductive Medicine Associates of New Jersey	Reprogenetics
Source	M. Katz-Jaffe^a^	J. Geraedts^a^	ISRCTN37972669^b^	NCT01194531^c^	NCT01219283^c^	NCT01332643^c^
Technology	SNP array	aCGH	aCGH	SNP array	qPCR	aCGH
Stage of biopsy	Blastocyst	Oocyte	Cleavage	Cleavage	Blastocyst	Blastocyst
Transfer type	Frozen	Fresh or Frozen	Fresh	Fresh	Fresh	Frozen
Major criteria for inclusion	>37 and/or ≥ 2 repeated IVF failures	36–40, <2 failed IVF cycles	36–43, <3 consecutive miscarriages, ≤ 2 failed IVF cycles	35–42, <3 consecutive miscarriages, ≤ 1 failed IVF cycle	21–43, day 3 FSH <15U/L, ≤ 1 failed IVF cycle	35–42, < 3 failed IVF cycles, day 3 FSH <11U/L
Estimated sample size	100	600	200	440	500	120
Estimated completion	January 2012	May 2013	January 2011	September 2011	July 2012	April 2012
Results	Not reported	Not reported	Not reported	Not reported	Scott et al.^25^	Not reported

^a^personal communication

^b^ www.controlled-trials.com

^c^ www.clinicaltrials.gov

## Future of CCS

While numerous considerations for the efficacy of using a specific stage of biopsy for PGS exist, one of the most important may be the impact that the procedure has on the reproductive potential of the embryo. Although some studies have focused on comparing outcomes in patients who had all of their oocytes/embryos biopsied to outcomes in patients who had none of their oocytes/embryos biopsied, this may not represent the best controlled study design. This is particularly true since the patients having embryos or oocytes biopsied were typically subject to PGS while the other patients were not. Instead, randomization of the 2 best oocytes/embryos so that one is biopsied (case) and the other is not (control) and subsequent paired analysis of outcomes within each 2 embryo transfer (using DNA fingerprinting) could represent a better controlled study of the impact of biopsy. Interestingly, preliminary results using this paired study design indicate that embryo biopsy at the cleavage stage, but not the blastocyst stage, significantly reduces the implantation potential of the embryo [74]. Similar studies of the impact of polar body biopsy have yet to be conducted but should be helpful in characterizing the safety and optimum stage of biopsy for CCS.

While validated methods of CCS may improve clinical outcomes for patients with infertility, they may also represent an exciting tool to help identify additional markers, beyond aneuploidy screening, that provide predictive information about the long term viability of embryos and oocytes. Indeed, this objective represents one of the most important challenges in reproductive medicine [75]. Since it is clear that not all embryos, identified as euploid by CCS, ultimately result in the delivery of a newborn, there are likely additional markers of reproductive potential that may enhance the precision of embryo selection. Once validated methods of CCS have been established, research efforts can be directed at characterizing molecular and biochemical signatures of embryos that are morphologically and chromosomally normal. One of the first studies to employ this design involved determining cumulus cells gene expression signatures predictive of which morphologically and chromosomally normal embryos possess reproductive potential [51]. This is different from simply evaluating whether cumulus cells can be used as an alternative to direct oocyte or embryo CCS aneuploidy screening [76] or whether cumulus cells can be used to select competent oocytes or embryos without simultaneous aneuploidy screening [77]. The unique aneuploidy controlled study design could apply to evaluating additional types of molecular signatures including, for example, the embryonic metabolome [78] or proteome [79, 80].

Despite the failure of cleavage stage FISH based aneuploidy screening, the introduction of new CCS technologies holds great promise for achieving the expected improvements in clinical outcomes for patients suffering from infertility. A critical mass of class I evidence still needs to be generated for each CCS methodology before scientists and clinicians adopt it for routine clinical testing. Randomized controlled trials of efficacy as well as non-selection studies of the negative predictive value of embryonic reproductive potential should provide the data needed to accept or reject the validity of a given CCS methodology. While recent success of blastocyst trophectoderm CCS with qPCR and fresh embryo transfer is encouraging, results from additional ongoing randomized trials of CCS are eagerly anticipated and should help shape the future of chromosome screening in the IVF setting.
